# Evaluation of the Linear Relationship Between Pulse Arrival Time and Blood Pressure in ICU Patients: Potential and Limitations

**DOI:** 10.3389/fphys.2018.01848

**Published:** 2018-12-21

**Authors:** Braiam Escobar-Restrepo, Robinson Torres-Villa, Panayiotis A. Kyriacou

**Affiliations:** ^1^City University of London, London, United Kingdom; ^2^Antioquia School of Engineering, Envigado, Colombia

**Keywords:** pulse arrival time, pulse tranist time, pulse wave velocity, blood pressure, intensive care unit

## Abstract

A variety of techniques based on the indirect measurement of blood pressure (BP) by Pulse Transit Time (PTT) have been explored over the past few years. Such an approach has the potential in providing continuous and non-invasive beat to beat blood pressure without the use of a cuff. Pulse Arrival Time (PAT) which includes the cardiac pre-ejection period has been proposed as a surrogate of PTT, however, the balance between its questioned accuracy and measurement simplicity has yet to be established. The present work assessed the degree of linear relationship between PAT and blood pressure on 96 h of continuous electrocardiography and invasive radial blood pressure waveforms in a group of 11 young ICU patients. Participants were selected according to strict exclusion criteria including no use of vasoactive medications and presence of clinical conditions associated with cardiovascular diseases. The average range of variation for diastolic BP was 60 to 79 mmHg while systolic BP varied between 123 and 158 mmHg in the study database. The overall Pearson correlation coefficient for systolic and diastolic blood pressure was −0.5 and −0.42, respectively, while the mean absolute error was 3.9 and 7.6 mmHg. It was concluded that the utilization of PAT for the continuous non-invasive blood pressure estimation is rather limited according to the experimental setup, nonetheless the correlation coefficient performed better when the range of variation of blood pressure was high over periods of 30 min suggesting that PAT has the potential to be used as indicator of changes relating to hypertensive or hypotensive episodes.

## Introduction

Arterial blood pressure is one of the four primary vital signs used as an indicator of the health status of the circulatory system that is commonly measured in clinical and ambulatory scenarios. The condition when blood pressure increases persistently above normal values (i.e., systolic above 140 mmHg) is called hypertension, a long term medical condition that is associated with coronary artery disease, stroke, heart failure and chronic kidney disease among others (Go et al., 2013; Judd and Calhoun, 2015). Continuous blood pressure monitoring is expected to improve hypertension detection and control especially in ambulatory scenarios, by providing rich information about fast and slow changes of blood pressure during the day supporting more optimally the management of disease for both the physician and patient. Nowadays there is a great variety of blood pressure monitoring devices using traditional methods, however, there is great demand for a system that is non-invasive, non-occlusive, unobtrusive, unsupervised, accurate, and continuous (Sola, 2011).

A variety of techniques based on the indirect measurement of blood pressure by Pulse Wave Velocity (PWV) have been proposed as a possible approach in meeting this gap and this is mainly due to the ability of the technique to perform beat to beat measurements non-invasively without the use of a cuff (Steptoe et al., 1976; Geddes et al., 1981; Chen et al., 2000). Pulse wave velocity is defined as the ratio between the length of the segment and the time it takes the pulse wave to travel from two arterial sites. When this time is measured from the electrocardiogram to a peripheral site, the cardiac pre-ejection period (PEP) is included and it is usually called Pulse Arrival Time (PAT), otherwise this time is regarded as Pulse Transit time (PTT). The theoretical relationship between PWV and blood pressure is commonly established by the combination of the Moens-Korteweg equation, that relates PWV with the elastic modulus and geometry of the artery (Wilmer Nichols, 2011), with the observations of Hughes that studied the exponential relationship of the elastic modulus with blood pressure (Hughes et al., 1979).

Many experimental studies have been reported over the last decades with the aim of evaluating whether these theoretical foundations can be applied in real settings and the spectrum of publications in the topic is broad. Studies include experimentation in animals and humans, central and peripheral arteries, comparisons with invasive and non-invasive blood values as reference and effects of vasoactive drugs among others. An excellent summary of the current state of the art was recently published by Mukkamala et al. (2015). One of the most important aspects about this set of publications is that the use of pulse arrival time as a marker of blood pressure outnumbers in great proportion those performed with pulse transit time. It seems reasonable to observe this trend considering the fact that PAT measured from the electrocardiogram (ECG) to the finger photoplethysmography (PPG) can be acquired non-invasively in clinical or experimental settings in a lab with ease, while pulse transit time can be more challenging to be obtained by non-invasive means, especially in regards to the proximal waveform.

Several authors have demonstrated that PAT is not an adequate surrogate of PTT for blood pressure estimation. Zhang et al. (2011) compared the ability of PAT and PTT measured from invasive arterial waveforms to track BP during induced changes by drug administration in six dogs. Their results showed that the root mean squared error (RMSE) between measured and predicted mean arterial pressure (MAP) was larger using PAT (10.4 ± 5.6 mmHg) compared to PTT (4.8 ± 1.0 mmHg). Payne et al. (2006) studied this phenomena in twelve healthy volunteers making beat to beat measurements of PAT (ECG to finger PPG), PEP (using cardiac bioimpedance), and PTT (PAT minus PEP). The overall coefficient of determination of the linear relationship between MAP and PAT was considerably low (*R*^2^ = 0.08) compared to PTT (*R*^2^ = 0.45). Both studies agreed about the influence of PEP as a confounding factor. In the contrary, other studies have elucidated the potential of PAT as a marker of blood pressure. Masè et al. (2011) reported an overall correlation *R*^2^ = 0.89 and 0.78 for systolic and diastolic BP, respectively with PAT in 33 healty subjects during increasing stress exercise. Gesche et al. (2012) carried out a study using a similar experimentation protocol with 63 volunteers and found a correlation coefficient *r* = 0.83 between PAT and systolic BP, although the predicted BP values were obtained using a non-linear model. He et al. (2013) and Choi et al. (2013) explored the capabilities of PAT in estimating blood pressure beat to beat in intensive care unit patients using the MIMIC database (Multiparameter Intelligent Monitoring in Intensive Care; Moody and Mark, 1996) and reported strong correlations with systolic BP (−0.92 and −0.721 for each publication). Although, the theory says that PTT is the most accurate timing reference rather than PAT, practical deviations by the use of the later have demonstrated some potential and the right balance between accuracy and convenience is still an unsolved problem worth exploring (Mukkamala et al., 2015).

The present study aims at exploring the degree of linear relationship between PAT and systolic and diastolic blood pressure in intensive care unit patients in an effort to provide further information about it capabilities and disadvantages in this particular scenario. The choice of an ICU population relies on the availability of a gold standard BP measurement (invasive waveform) as a reference and the potential to assess its relationship with PAT over long periods of time under controlled settings. It has been reported that the relationship between these variables deteriorates over time, and periodic recalibration of the parameters of the regression model is needed (Poon et al., 2008; Cattivelli and Garudadri, 2009). Considering that, the present work analyzes the relationship between PAT and BP for a group of ICU patients individually in two ways: an overall correlation coefficient is provided for the entire recording time in each patient while the dynamic change of such relationship over time is analyzed by performing the correlation over consecutive segments of shorter duration.

## Methods

### Study Population

Eleven patients aged 18 to 50 (7 males - 4 females) from the medical ICU of the Hospital Universitario de San Vicente Fundacion in Colombia were recruited over a period of 6 months for a total of 96 h of recordings of their physiological waveforms. Candidates for the study were selected according to strict exclusion criteria that comprised assisted mechanical ventilation, vasopressors, and vasodilators use prior or during the acquisition time, scheduled surgical procedure within the next 24 h and clinical history with previous cardiovascular diseases in their lifetime. Permission for data acquisition was granted by the Ethics committee of Universidad EIA and Hospital Universitario de San Vicente Fundacion.

### Signal Acquisition System

Physiological waveforms from the patients were acquired using the Philips MP40 IntelliVue monitors available at each ICU bed. Through a communication interface protocol, data from the Philips monitor can be transferred via the Local Area Network (LAN) or Medical Information Bus (MIB/RS232) Interfaces to an external computer (Philips, 2011). The LAN interface was chosen for data acquisition and an application was developed in Matlab^®^ to communicate the bedside monitor with a laptop computer over the standard UDP/IP protocol. Acquisition started immediately after the patient was included in the study and stopped only if any of the exclusions criteria were violated during the recording time (i.e., administration of vasopressor drug) or the patient was discharged from the ICU. The invasive arterial blood pressure (ABP) waveform at the radial artery of either arm was obtained at 125 Hz while the Electrocardiography (ECG) lead II featured a 500 Hz sampling rate.

### Data Processing

**Figure 1:**
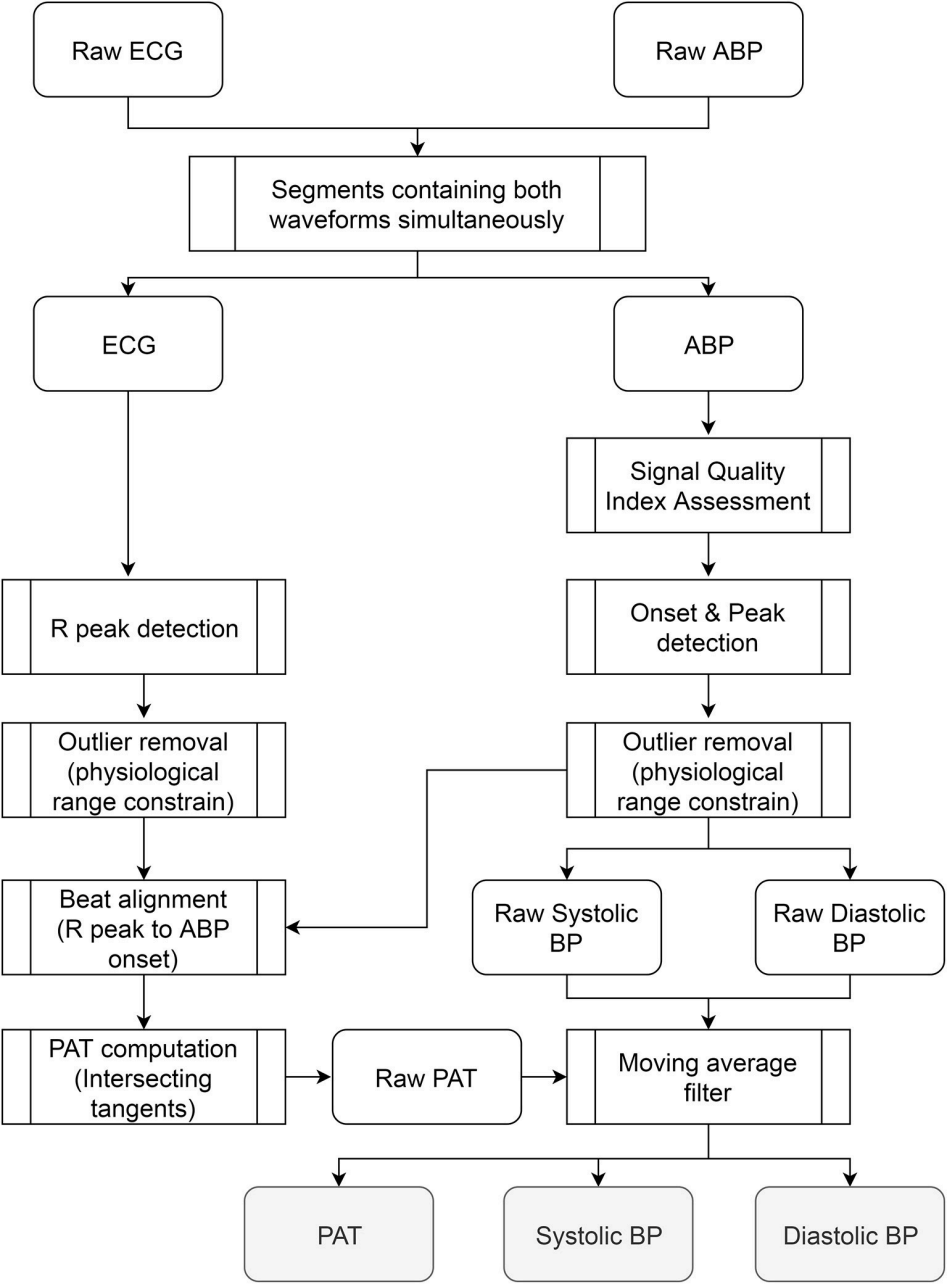
Overall view of the processing stages carried out in the database. The steps were applied to the waveforms recorded from each patient independently. The outputs are three vectors (PAT, systolic, and diastolic BP) whose size depends on the number of beats available in the recording.

Figure 1 presents an overall view of the processing stages carried out in the database. Since both waveforms were continuously acquired from the subjects without special restrictions in order to facilitate the normal activities of nurses and healthcare professionals, several time sections of the database were not able to provide all waveforms simultaneously or exhibited excessive motion artifact noise. Scenarios like having meals, receiving a visit from family, taking a shower, and sensor cleaning or replacement hindered the correct estimation of pulse arrival time during certain passages. To obtain a clean dataset for the study only intervals that simultaneously contained ABP and ECG were included keeping their original time stamps so no concatenation of segments was performed to preserve the time reference.

The quality of the ABP waveform was assessed in Matlab^®^ using a signal quality index based on beat template correlation proposed by Li and Clifford (2012). The algorithm introduces a dynamic time warping to stretch each ABP beat to match a running template and combines it with several other features related to signal quality, including correlation and the percentage of the beat that appeared to be clipped, resulting in an index of quality between 0 and 1.This algorithm was applied across all subjects in segments of 5 min with no overlapping and pulses that exhibited an index below 0.9 were excluded from the analysis. Onsets on the ABP waveform were identified using the WFDB Toolbox for MATLAB available at Physionet (Goldberger et al., 2000). The detection algorithm (called wabp) proposed by Zong et al. (2003) is based on analysis of the first derivative on the waveforms and outputs an annotation file in which all detected beats are labeled normal. Once the onsets on the ABP were obtained, values of systolic, diastolic, and mean blood pressure were identified and another quality assessment algorithm (called jSQI) proposed by Sun et al. (2004) was applied to supress possible outliers or mistakes in previous processing. Sun's algorithm relies on detecting abnormalities of numeric values that diverge from physiological conditions (i.e., systolic beat-to-beat change >20 mmHg).

The QRS complexes in the ECG waveform were identified using a batch Matlab^®^ function developed by Clifford (2006) and made available under the GNU general public license. The algorithm is based on the original Pan and Tompkins implementation from 1985 which relies upon digital analyses of slope, amplitude, and width of the ECG (Tompkins and Pan, 1985). Heart rate was calculated from the complexes and the reliability of the estimates was assessed by imposing a valid range of variation between 20 and 200 bpm according to normal physiological conditions.

**Figure 2:**
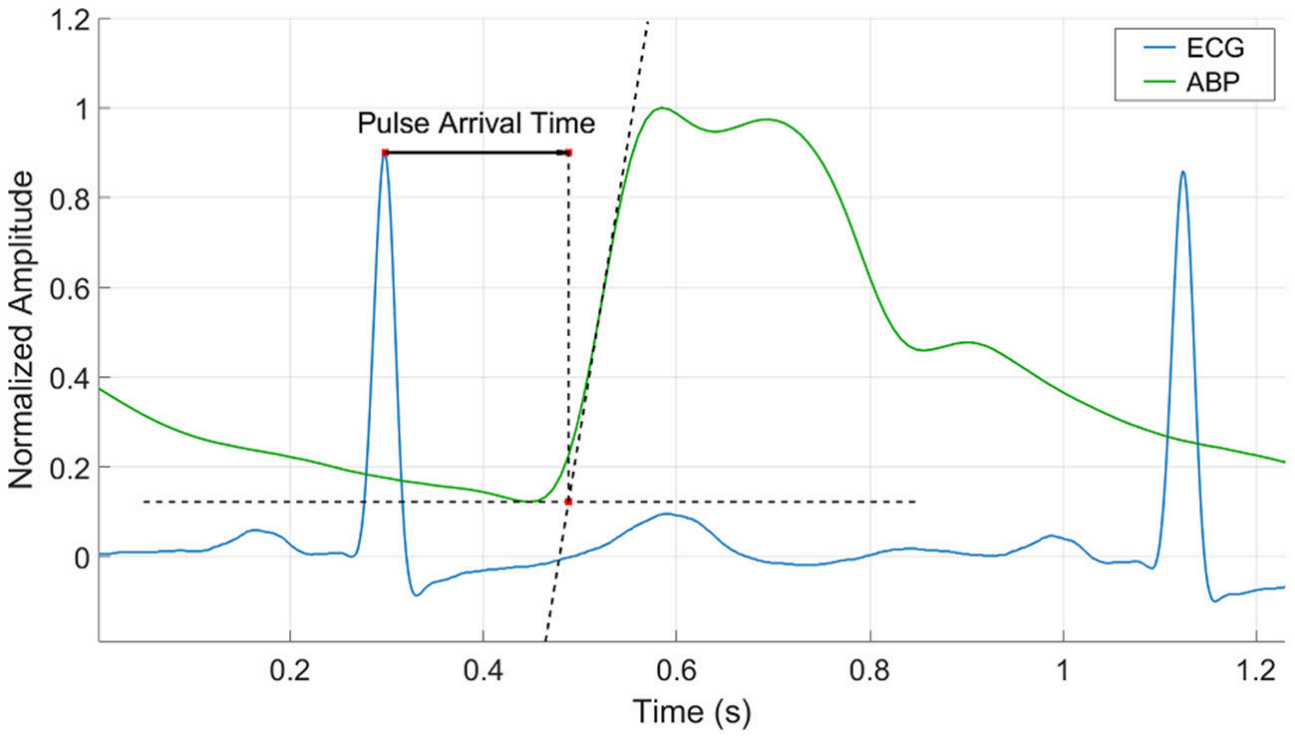
Pulse Arrival Time calculation by the intersecting tangent method. The time is estimated from the r-peak of the ECG waveform to the intersection point of the tangent to the maximum gradient and tangent of the onset value in the ABP waveform.

Pulse Arrival Time was estimated beat to beat for all subjects. The R-peak on the ECG was used as the starting time reference and was matched with its corresponding feature on the ABP waveform. This feature was calculated based on the “intersecting tangents” that is the intersection point of the tangent to the maximum gradient and tangent of the onset value in the waveform (Figure 2). This method has been recognized as a robust marker in the distal waveform in previous pulse transit time studies since the influence of wave reflections is attenuated and is less disturbed by noisy pulses (Davies and Struthers, 2003; Zhang et al., 2011; Hemon and Phillips, 2016).

It is well known that many cardiovascular parameters such as blood pressure, heart rate, and pulse transit time exhibit a beat to beat variability that contain components in different frequency bands which have been associated with autonomic regulatory mechanisms (Ma and Zhang, 2006; Ma et al., 2016). Since the main objective is to assess the level of linear correlation between pulse arrival time and blood pressure, these beat to beat variations were attenuated as they could play a role in the correlation, considering that their amplitude and phase coupling were not evaluated in the present work. A median moving average filter on a window of 30 s overlapped every 7.5 s was applied to the raw systolic, diastolic and pulse arrival time vectors for each subject. A minimum of 15 beats were required in the 30 s window for the median calculation to be valid, otherwise the result was marked as NaN (Not a Number) so the timing reference in the vectors is kept but that specific segment of data is not considered in the subsequent analysis.

### Data Analysis

The degree of linear relationship between pulse arrival time and blood pressure was assessed in two perspectives. First, the Pearson correlation coefficient (CC) and Mean Absolute Error (MAE) were calculated for each subject independently to elucidate the overall relationship between BP and PAT in the entire recording time. The ratio between MAE and the variation range of blood pressure was estimated and compared for all subjects. The Mean Absolute Error (MAE) was computed according to equation 1. A second analysis was performed to evaluate how the correlation coefficient changes over time in order to assess the dynamic variation of the relationship between these variables. For this purpose, the CC between PAT and BP was estimated on windows of 30 min with no overlap and the overall results for all subjects are presented. Both analyses were done for systolic and diastolic blood pressure independently and their standard deviations were observed and compared with the corresponding correlation coefficient.

$$\begin{array}{l} MAE=\;\frac{\sum_{i=1}^{n}|PE_{i}-\;PO_{i}|}{n} \end{array}$$

Equation 1. Mean Absolute Error (MAE) computation. *PO is* the observed pressure (derived from the invasive arterial waveform) and *n* the total number of observations in the subject's recording. *PE* is the estimated Pressure using PAT. The linear regression parameters to compute PE were obtained using all the BP measurements available per subject.

## Results

Of the initial 96 h of recordings from all subjects, 23 were excluded from the analysis because both ECG and ABP were not present simultaneously or the waveforms in certain sections did not meet the signal quality threshold, so a total of 73 h of data were analyzed containing 333,007 beats. Table 1 shows the Pearson correlation coefficient and mean absolute error after assessing the relationship between PAT and systolic/diastolic blood pressure for each subject. To describe the overall CC for the study, the mean value for all subjects is presented as the arithmetic average of each column (diastolic CC = −0.46, systolic CC = −0.56) and the adjusted mean (diastolic CC = −0.42, systolic CC = −0.52) representing the weighted average by considering the number of beats analyzed. The table also describes the range of variation of each variable (MAX, MIN and Δ = MAX-MIN) with diastolic blood pressure varying in average from 60 to 80 mmHg, systolic blood pressure from 123 to 158 mmHg and pulse arrival time from 174 to 200 ms which are within a normal physiological range expected for ICU patients. The mean absolute error for the diastolic BP did not surpassed 5 mmHg for any of the subjects having an adjusted mean of 3.86 (± 1.28) mmHg. The MAE for the systolic BP was generally higher compared to the diastolic one showing an adjusted mean value of 7.59 (± 3.17) mmHg and reaching 12.37 mmHg for subject #1. The lowest MAEs for the systolic (subjects 4, 6, 7, and 11) are associated with lower BP variation ranges (29, 14, 16, and 15 mmHg). The ratio between MAE and the BP range of variation (Δ) showed a similar and consistent trend (mean = 0.16, adj mean = 0.17) for both systolic and diastolic BP with standard deviations less than ± 0.04.

**Table 1:** Summary of results detailed for each subject.

**Subject**	**Beats**	**PAT (ms)**			**Diastolic BP (mmHg)**						**Systolic BP (mmHg)**					
		**MAX**	**MIN**	**Δ**	**CC**	**MAE**	**MAX**	**MIN**	**Δ**	**MAE/Δ**	**CC**	**MAE**	**MAX**	**MIN**	**Δ**	**MAE/Δ**
1	73,910	230	184	46	−0.54	5.00	84	51	33	0.15	−0.59	12.37	168	99	69	0.18
2	58,363	191	160	31	−0.62	4.03	95	70	24	0.17	−0.32	8.84	198	149	49	0.18
3	80,199	186	153	33	−0.10	4.95	78	55	23	0.21	−0.55	6.32	176	143	33	0.19
4	7,311	232	196	36	−0.89	2.35	96	72	24	0.10	−0.92	2.61	160	131	29	0.09
5	18,691	221	206	15	−0.63	1.96	86	73	12	0.16	−0.26	7.48	186	148	38	0.20
6	15,510	187	173	13	0.16	1.64	71	62	9	0.18	−0.32	2.24	141	128	14	0.16
7	13,575	222	199	24	−0.13	2.32	59	46	13	0.17	−0.65	2.48	127	110	16	0.15
8	4,721	161	140	21	−0.43	4.17	92	71	21	0.20	−0.43	7.54	162	121	41	0.18
9	40,926	224	192	32	−0.53	2.50	64	48	16	0.16	−0.54	6.24	142	103	40	0.16
10	10,435	165	148	17	−0.43	4.12	85	63	22	0.19	−0.67	5.90	158	120	39	0.15
11	9,366	184	166	18	−0.96	0.80	59	47	12	0.07	−0.93	1.35	116	101	15	0.09
Mean	30,273	200	174	26	−0.46	3.08	79	60	19	0.16	−0.56	5.76	158	123	35	0.16
std (±)	28,180	26	23	10	0.335	1.425	14	11	7	0.043	0.227	3.353	25	19	16	0.037
Adj Mean*		205	173	32	−0.42	3.86	80	57	23	0.17	−0.52	7.59	168	125	43	0.17
std (±)		21	18	9	0.26	1.28	11	9	7	0.03	0.15	3.17	21	21	17	0.02

*PAT, diastolic, and systolic ranges are shown along with the Pearson correlation coefficients (CC) and mean absolute error (MAE) as resulted from the linear regression*.

* *Adj Mean. Represents the weighted average of the corresponding column taking into account the proportion of beats for each subject*.

**Figure 3:**
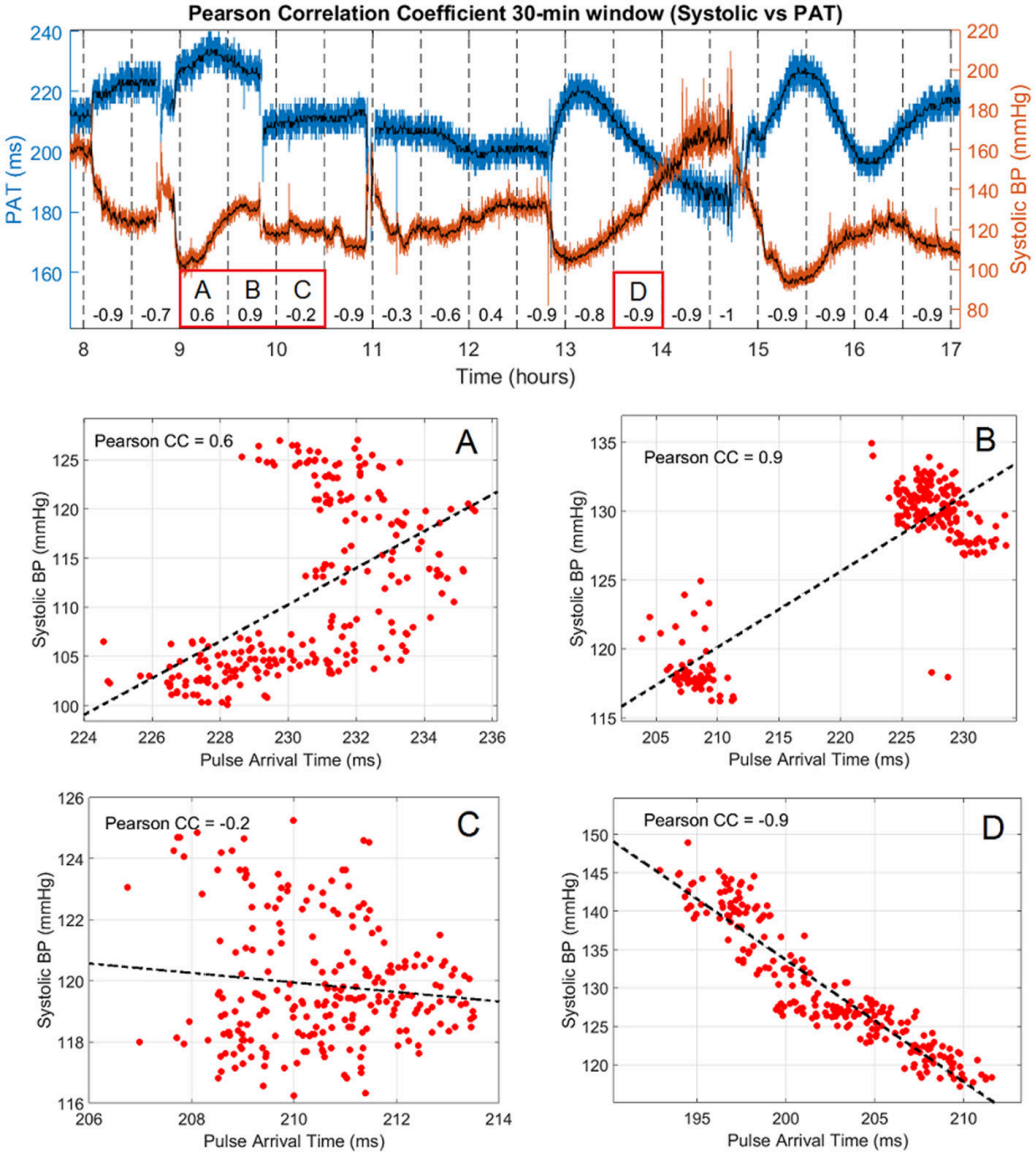
Pearson correlation coefficient and systolic vs. PAT variation in 9 h of subject #1. Raw values of PAT and systolic BP are shown in blue and red, respectively with the corresponding median filtered values in black. The correlation coefficient is presented every 30 min in the lower part of the graph and the PAT vs. Systolic BP dispersion of 4 segments of interest are labeled **(A–D)**.

The second part of the analyses assessed how the Pearson correlation coefficient between PAT and BP varied over time in windows of 30 min for each subject. An example of the systolic-PAT variation for 9 h of recording in subject #1 with the corresponding correlation coefficient at each window is shown in Figure 3. It can be seen how the correlation coefficient varies almost in the full range (from 0.9 to −1) in that period. Four cases are labeled in Figure 3 where: (A) shows a period in which almost both PAT and BP have an increasing trend so the CC is positive. (B) depicts a sudden change in PAT and BP and both variables took the same direction forming two clusters that can be seen on the dispersion graph producing a positive CC. Although the Pearson correlation coefficient is not well suited for assessing relationship in this type of distribution, it accounts for their great magnitude change on the same direction. In (C) the correlation is negative but weak, with the systolic BP varying only 10 mmHg and PAT around 7 ms. (D) shows a strong negative correlation between the variables with a wide variation in their range.

**Figure 4:**
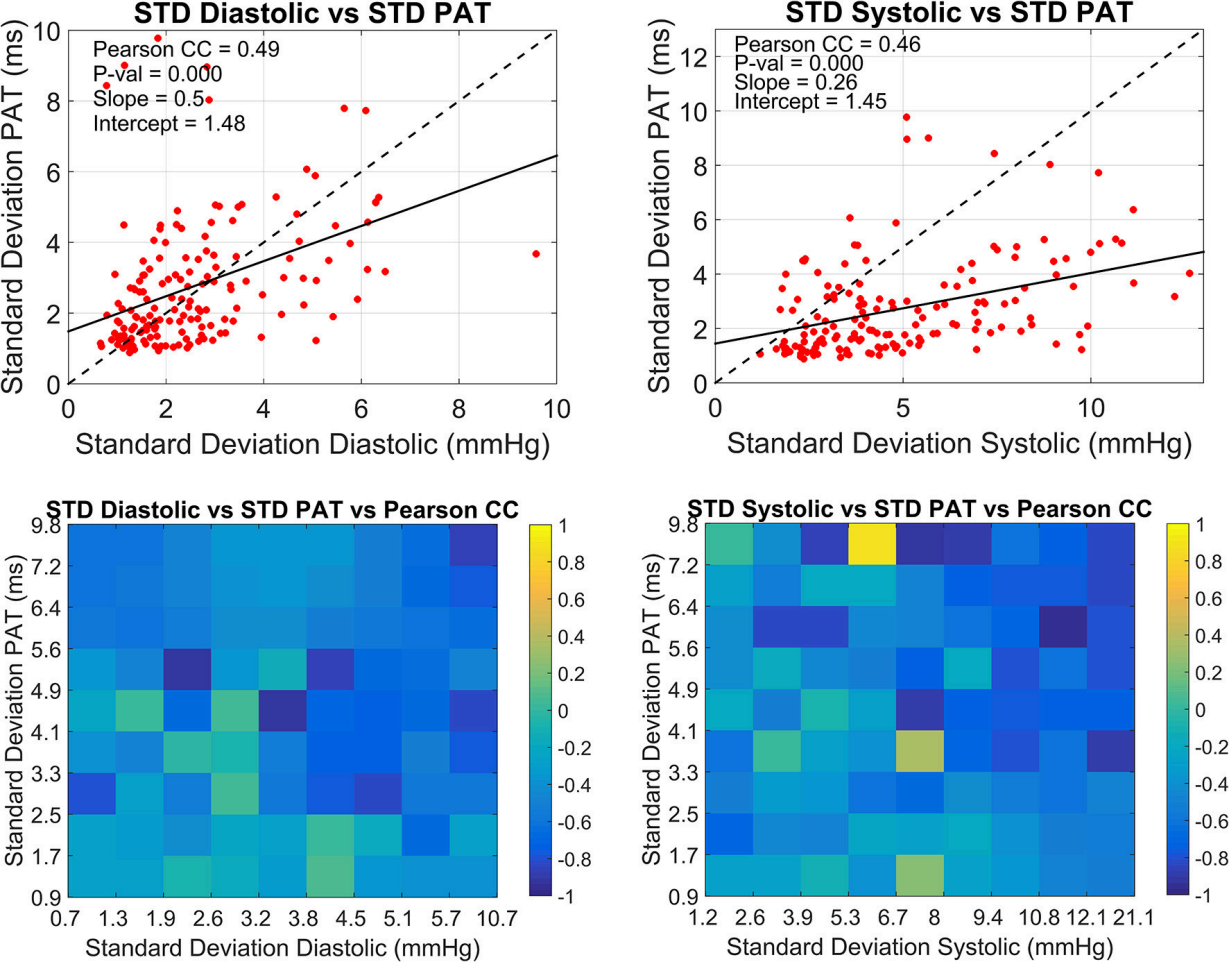
Overall relationship between the standard deviations of PAT and BP with the correlation coefficient. The dispersion between the standard deviation of PAT and the standard deviation of diastolic BP (left) and systolic BP (right) calculated every 30 min are presented in the upper side. Lower plots show the corresponding correlation coefficient for each upper plot.

The overall relationship between the standard deviations of PAT and BP with the correlation coefficient in windows of 30 min for all subjects is summarized in Figure 4. The upper graphs depict the relationship between the standard deviation of PAT and the standard deviations of diastolic and systolic BP correspondingly. The slopes of the fitted lines are 0.5 ms/mmHg for the diastolic and 0.26 ms/mmHg for the systolic. The lower graphs extend this analysis by including the correlation coefficient found for the BP-PAT relationship detailing how small and large variations in BP and PAT are linked to high or low CC. This correlation coefficients tend to be more negative (darker blue) in the upper right corner of each box where the standard deviation of both PAT and BP is higher, being more prominent in the systolic graph.

## Discussion

The present study analyzed the degree of linear relationship between systolic/diastolic blood pressure and pulse arrival time in a total of 96 h of ICU waveforms from 11 subjects. The choice of strict exclusion criteria was justified in order to obtain a subset of patients exhibiting slightly different characteristics than the average ICU patient that allowed exploring the relationship of the variables in a different fashion. Specifically, the age was restricted from 18 to 50 years since usually the majority of these patients are elderly which is associated with increased arterial stiffness (Wilmer Nichols, 2011) and although it was not measured for each patient, the prevalence of more compliant arteries in the cohort was favored. Another important aspect is the exclusion of recordings in which patients were treated with vasopressors and vasodilators. It would have added more complexity to the comparison across patients considering the heterogeneity of drugs, doses, and time of administration between them. Also, the requirement of no previous or current admission for cardiovascular diseases aimed in achieving a more homogeneous population, minimizing the influence of more confounding factors but noticeably reducing the number of participants to 11.

Another aspect worth commenting is the exclusion of the PPG waveform from the study. Vital sign monitors frequently apply strong filtering stages to the raw PPG signal in order to minimize its baseline wandering and to improve visualization on the display. It is not known exactly whether the PPG signal obtained from the Philips MP40 monitor provided the raw signals nor the information about the LED selected (red or infrared). Pulse arrival time calculated from the ECG to the onset of this signal resulted in values around 400 to 500 ms, which do not match with the expected range reported in previous studies (Buxi et al., 2015; Mukkamala et al., 2015). It was also observed that the PAT followed a saw tooth pattern with a period around 20 s which might be associated with additional clocking issues. Regarding the invasive arterial waveform, filtering stages tend to be less strong since the actual systolic and diastolic values have to remain unaffected. No saw tooth pattern was observed on the PAT calculated when using this waveform and its variation was according to the expected physiological range.

Robust signal processing methods play an important role when assessing the degree of relationship between the variables, since misattribution of detected features (i.e., peaks or onsets) or alignment issues when estimating PAT (incorrect matching of R-peak to PPG onset) tend always to blur the correlation instead of improving it. The choice of well-known algorithms for calculating the points of interest on each waveform and the use of signal quality indexes are considered an important asset of the present study. As described in the methodology, the raw PAT and BP vectors were filtered using a median moving average filter with a window of 30 s overlapped every 7.5 s. This filter has the advantage of suppressing remaining outliers in the data while providing an even resampled vector that does not depend on the subject's specific heart rate. The selection of such average filter is also in accordance with common monitoring practices considering that vital signs monitors usually display blood pressure estimates as averaged values over short periods of time.

The results show that the correlation between pulse arrival time and blood pressure is within a moderate to low range in general. Systolic BP performed better than diastolic BP in terms of the Pearson correlation coefficient (−0.52 vs. −0.42). Although the experimental setup is not identical, our results are in agreement with previous studies that have observed a similar trend using invasive arterial waveforms for the analysis (Spulak et al., 2011; Escobar and Torres, 2014; Gao et al., 2016). Douniama et al. (2009) reported an investigation of blood pressure tracking capabilities of pulse transit times in different arterial segments in 22 sedated ICU patients. PTT in the subject's arm was measured in several ways by combining ECG, PPG, ICG (impedance cardiogram), IPG (impedance plethysmogram), and radial ABP. Of all the possible combinations the PTT measured from the ECG to the radial ABP performed the best though the correlations coefficients were low (0.47 for systolic and 0.42 for diastolic) and in agreement with our findings.

In terms of the overall adjusted Mean Absolute Error, the systolic BP reached nearly 8 mmHg while the diastolic half that amount. These two quantities might seem encouraging for a technique trying to estimate BP continuously by PAT, however the analysis of this result has to be carried out in the context of the corresponding BP range of variation. In this study an overall ratio of 0.17 was found by dividing MAE over the BP range for each subject, meaning that for example an increase of 10 mmHg in the systolic BP would generate an additional error of 1.7 mmHg in the estimated BP using PAT. If one translates this result to the grading standards for medical devices of the Association for the Advancement of Medical Instrumentation (AAMI) it would imply that a system measuring BP by PAT is classified grade A only if during validation the BP does not change on a range >29 mmHg (5/0.17). This is in fact a narrow range for normal physiological conditions exposing the weaknesses of the technique.

The temporal change of the Pearson correlation coefficient was also assessed in the present work. The motivation relies in previous studies that have showed how the relationship degrades over time and periodic recalibration techniques have to be implemented to maintain the accuracy within acceptable limits (Cattivelli and Garudadri, 2009; McCarthy et al., 2013). It is common to see correlations between these variables in periods ranging from minutes to hours but to the best of our knowledge very few investigations have been conducted to explore its dynamic change over time. For this purpose an arbitrary window of 30 min was selected with no overlap to analyse its behavior during the recording time for all subjects. It can be inferred from the graphs presented in Figure 4 that the best scenario for a high correlation between the variables is when BP has a wide variation while on the contrary is more affected in periods of low variation. This observation could be explained by considering the already known contribution of the cardiac pre-ejection period on PAT which is typically around 50 to 100 ms (Payne et al., 2006; Wong and Zhang, 2008). For this study the mean PAT across subjects was 200 ms meaning that PEP could probably be contributing for up to 50% of the measurement. It could be hypothesized that in cases where BP had pronounced swings in the 30 min window, the variation of transit time was higher in absolute units compared to the PEP variation, so the strength of the trend is mainly driven by the transit time and BP. For cases of low BP variation it is expected that the change in transit time is also low but even minor changes in PEP could blur the correlation. This hypothesis unfortunately cannot be tested in this work due to the lack of PEP measurements. If this analysis is translated to a more general ICU context where patients are treated with vasopressor drugs for example, the contribution of PEP would be more challenging to infer and the current reasoning might not be as simple as described. This is also due to the fact that vasoactive drugs modify the diameter, thickness and elastic modulus of most of the arterial tree involved in the PAT estimation so a straight forward relationship between BP and PAT even in cases of broad changes would be questionable.

Another limitation of the analysis presented in the current work is the imbalanced number of cases over the BP range of variation since more cases are found in the lower range. A future study in a larger database like MIMIC would allow an even analysis by providing cases over a balanced range of BPs.

## Conclusion

The present study demonstrates that the degree of linear relationship between blood pressure and pulse arrival time measured from the ECG to the invasive arterial waveform in a small cohort of ICU patients is within a moderate to low range, hence the ability of PAT for continuous non-invasive blood pressure estimation is questioned. Despite this, it was observed that PAT correlates better with BP when the later exhibits wide variations in short periods, so PAT might have some potential as an indicator of wide trend changes like in hypertensive or hypotensive episodes occurring in this type of patients.

## Author Contributions

BE-R, RT-V, and PK contributed conception and design of the study. BE-R acquired and organized the database. BE-R performed the statistical analysis. BE-R wrote the first draft of the manuscript and prepared tables and figures. All authors contributed to manuscript revision, read and approved the submitted version.

### Conflict of Interest Statement

The authors declare that the research was conducted in the absence of any commercial or financial relationships that could be construed as a potential conflict of interest.
